# OptoH_3_R fusion protein mimics β-arrestin-mediated membrane endocytosis of histamine H_3_ receptor in vitro

**DOI:** 10.1186/s13041-026-01322-1

**Published:** 2026-06-20

**Authors:** Shuhui Deng, Zhuchen Zhou, Zhong Chen, Yanrong Zheng, Jing Zhou

**Affiliations:** Zhejiang Collaborative Innovation Center for the Brain Diseases with Integrative Medicine, Zhejiang Key Laboratory of Neuropsychopharmacology, School of Pharmaceutical Sciences, Zhejiang Chinese Medical University, Hangzhou, Zhejiang China

**Keywords:** OptoH_3_R, β-arrestin pathway, Optogenetics, Cell signaling, Histamine H_3_ receptor

## Abstract

OptoH_3_R is an artificial fusion protein that combines the photosensitive elements of rhodopsin with the signaling domain of the histamine H_3_ receptor (H_3_R), enabling light-controlled activation of downstream H_3_R pathways. Although our previous study demonstrated that OptoH_3_R mimics the acute effects of H_3_R activation on neuronal activity, whether this tool can also recapitulate the long-term receptor desensitization and internalization processes associated with prolonged H_3_R activation remains unclear. In this study, primary cortical neurons and HeLa cells were employed to investigate the alterations in subcellular localization of OptoH_3_R upon sustained photoactivation, with histamine-stimulated wild-type (WT) H_3_R serving as a control. Furthermore, the role of β-arrestin in this process was explored. Time-lapse fluorescence imaging revealed that the number of puncta progressively increased over time following laser stimulation. Subsequent co-staining experiments with endosome marker EEA1 showed that 75.5% of light‑induced puncta were EEA1‑positive. Notably, the increase in OptoH_3_R-positive vesicles within neuronal cells was attenuated by the β-arrestin inhibitor barbadin, a pattern consistent with the internalization observed in histamine-stimulated WT H_3_R. Collectively, our findings demonstrate that OptoH_3_R recruits β-arrestin signaling upon sustained optical stimulation, thereby recapitulating H_3_R desensitization dynamics. This establishes OptoH_3_R as a useful tool for dissecting the spatiotemporally specific functions of H_3_R, including both its acute signaling and long-term β-arrestin-related mechanisms.

Optogenetics uses light-sensitive proteins to precisely manipulate specific neurons [1]. However, its limitations in elucidating the precise contributions of individual protein molecules to physiological and behavioral outcomes have spurred the emergence of optopharmacology, which employs photosensitive molecules to directly modulate endogenous proteins (e.g., ion channels, GPCRs) [2, 3]. Among these, OptoXRs, engineered from rhodopsin, enable optical control of intracellular signaling, even in behaving animals [4].

The histamine H_3_R plays a critical role in the central nervous system (CNS) by modulating the release of histamine and other neurotransmitters [5]. Emerging evidence indicates that histamine receptor functions are highly cell-type specific, and their downstream signaling encompasses both G protein-dependent pathways and β-arrestin-mediated regulatory mechanisms [6]. OptoXRs technology offers a powerful means to investigate such cell-type-specific signaling by enabling precise spatiotemporal control of receptor activation [7]. We previously demonstrated that OptoH_3_R (a photosensitive histamine H_3_R fusion protein) successfully engages downstream G protein pathways and mimics the acute effects of H_3_R activation on neuronal activity [8]; however, its role in β-arrestin-mediated signaling and long-term regulation—including receptor internalization and subsequent desensitization—remains unexplored and requires further investigation.

In this study, we assessed whether sustained OptoH_3_R activation recruits β-arrestin and induces internalization, thereby further validating its utility as a precision tool for receptor research. The OptoH_3_R was constructed by fusing the domain fusion of bovine rhodopsin and human H_3_R. Specifically, the extracellular and transmembrane domains of rhodopsin are retained for photosensitivity, while its intracellular loops and C-terminus are replaced with corresponding functional regions of H_3_R (based on Uniprot Q9Y5N1 annotations). A short rhodopsin-derived sequence (TETSQVAPA) is added to the C-terminus to ensure proper folding and localization. In addition, the constructed OptoH_3_R is expressed via a Cre-dependent adeno-associated virus (AAV-CAG-FLEX-OptoH_3_R-EGFP, kindly provided by Professor Xuhua Wang from Zhejiang University). This OptoH_3_R chimeric receptor integrates rhodopsin’s light-sensing properties with the intracellular signaling capabilities of the H_3_R, enabling optical control (488 nm laser stimulation) of H_3_R-mediated downstream signaling pathways. The photosensitive module consists of the rhodopsin (Bos Taurus, NM_001014890) transmembrane domain, which undergoes conformational changes upon illumination at specific wavelengths. The intracellular domain of the histamine H_3_R (Homo sapiens, NM_007232.3) serves as the signal transduction element and is fused directly to the rhodopsin transmembrane region. Specifically, OptoH_3_R retains the extracellular and transmembrane domains of rhodopsin (Fig. 1a), which provides light sensitivity and removes native H_3_R ligand-binding pockets, while incorporating the intracellular loops of H_3_R to preserve specific downstream signaling functionality. This configuration allows light-induced activation of H_3_R signaling pathways without the need for endogenous histamine binding.

To investigate whether the OptoH_3_R can mimic endogenous histamine H_3_R activation of its downstream β-arrestin signaling pathways, primary mouse neuronal cells were transfected with AAV-FLEX-OptoH_3_R-EGFP and AAV-hSyn-Cre-IRES-mCherry. After 72 h transfection, cells were illuminated with a 488 nm laser (Olympus IX83-FV3000 confocal, Coherent OBIS 488 nm LS 20 mW CW, 80% laser power) for 10 min per imaging region and observed using the confocal microscope, in contrast to the acute 30‑s stimulation typically used for rapid signaling events, to allow sufficient time for receptor internalization to occur. Using histamine-stimulated WT H_3_R as a control, OptoH_3_R-positive vesicles exhibited internalization patterns similar to that of WT H_3_R, including comparable puncta number per cell (7.333 ± 0.8819 for light-stimulated OptoH_3_R vs. 8.4 ± 0.5583for histamine-stimulated WT H_3_R after 1 h) and subcellular distribution (Fig. 1d and f), which were both attenuated by barbadin, a β-arrestin inhibitor (Fig. 1b-e, *p* < 0.001). Furthermore, the time-lapse imaging was conducted in living HeLa cells expressing OptoH3R-EGFP to monitor the dynamic changes of these vesicles within subcellular structures over 1 h (with imaging every 10 min), and an increase in the number of puncta over time was observed (Fig. 1f-g). Subsequently, these cells were co-stained with an early endosome marker, rabbit anti-EEA1 (1:100; Cell Signaling, 3288T, USA), which revealed a high degree of colocalization (75.5%) between the vesicular puncta and endosomes (Fig. 1h). This finding indicates that the light-induced increase in OptoH_3_R puncta represents receptor endocytic vesicles. Taken together, these results demonstrate that OptoH_3_R can mimic the activation of H_3_R and its downstream β-arrestin signaling, including acute G protein-mediated effects and β-arrestin-dependent regulatory processes.

**Fig. 1:**
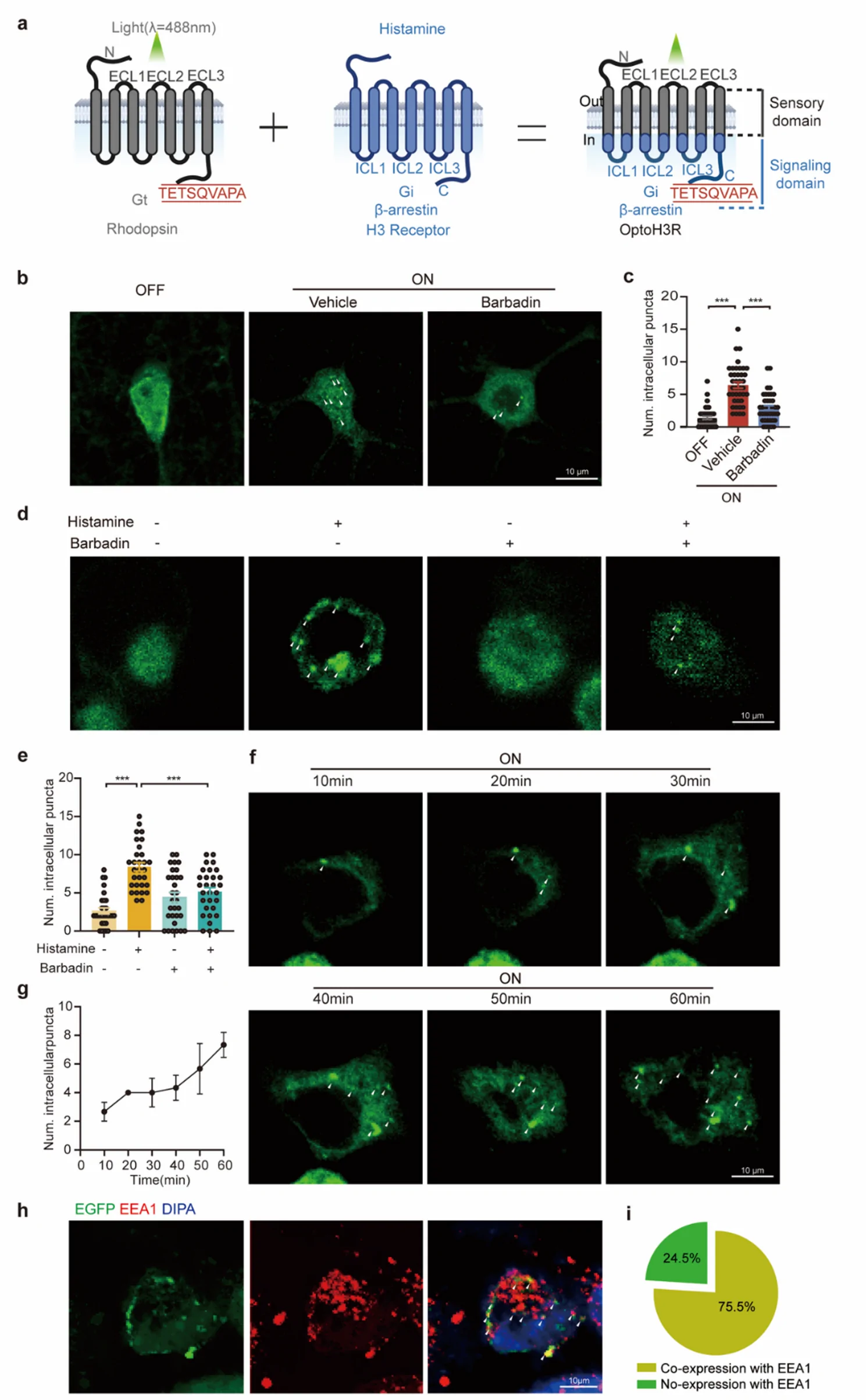
Design of OptoH_3_R and its application in simulating the endocytosis of β-arrestin pathway. (a) Schematic diagram of the OptoH_3_R design [8]. (b) The primary neuronal cells are transfected with AAV-FLEX-OptoH_3_R-EGFP and AAV-hSyn-Cre-IRES-mCherry for 72 h, and pre-incubated with DMSO (vehicle) or barbadin (100 μM) for 30 minutes, followed by exposure or no exposure to 488 nm laser light (80% laser power) for 10 min per imaging field. Representative images of EGFP fluorescence; (c) the number of intracellular EGFP-positive puncta per cell. n=40 cells per group. Scale bar, 10 μm. (d) The endocytosis image of WT-H_3_R under histamine stimulation. The wild-type HeLa cells are transfected with H_3_R-EGFP for 24 h, and pre-incubated with DMSO (vehicle), barbadin (100 μM) for 30 minutes or histamine (100 μM) for 1 hour. Representative images of EGFP fluorescence; (e) the number of intracellular EGFP-positive puncta per cell. n=30 cells per group. Scale bar, 10 μm. (f) Time-lapse imaging reveals dynamic internalization of OptoH_3_R upon photoactivation over 1 hour. Time-lapse imaging was performed in a 37°C, 5% CO environmental chamber over 1 hour. Representative fluorescence images captured at 10-minute intervals. Scale bar, 10 μm; (g) the number of intracellular EGFP-positive puncta at each time point (n=3 cells from three independent experiments). (h) OptoH_3_R puncta co-localize with the early endosomal marker EEA1 (n=106 puncta from 20 cells). Scale bar, 10 μm. All experiments were performed in triplicate independently. Data were analyzed using one-way ANOVA followed by Tukey’s multiple comparison test and are presented as means ± SEM. *** p < 0.001 versus the indicated group

As a key CNS receptor involved in fear memory, cognition, and other physiological processes, the functional interpretation of the H_3_R has long been constrained by the inherent limitations of traditional research methods, leading to substantial contradictions across different studies [6, 9]. These apparently paradoxical findings underscore the critical importance of spatial specificity in H_3_R function: receptor activity in different brain regions, even within distinct neuronal populations in the same region, may produce opposing behavioral outcomes. Beyond spatial complexity, the temporal dynamics of H_3_R signaling add another layer of intricacy [10]. Our previous study demonstrated that OptoH_3_R faithfully mimics H_3_R-mediated G protein signaling and the acute effects on neuronal activity [8]. The present findings extend this observation by demonstrating that OptoH_3_R undergoes internalization in response to sustained light stimulation. The blockade of this phenomenon by a β-arrestin inhibitor indicates the specific involvement of the β-arrestin pathway in this internalization process. The ability of OptoH_3_R to replicate both arms of H_3_R signaling—G protein-mediated effects [8] and β-arrestin-dependent internalization—highlights its utility as a precise optogenetic tool for dissecting spatiotemporally specific functions of H_3_R. Moreover, the successful use of OptoH_3_R in primary mouse neurons expressing Cre recombinase (via AAV-FLEX and AAV-hSyn-Cre) underscores the versatility of this approach for cell-type-specific manipulations.

However, it is worth noting that the present study employed global illumination of entire imaging regions; future experiments incorporating spatially restricted light patterns could reveal whether localized OptoH_3_R activation is sufficient to drive endocytosis or whether widespread receptor engagement is required. Another limitation is the use of relatively high laser power (80%), which raises the possibility of phototoxicity or off-heating effects. Additionally, this study was conducted exclusively in vitro, whether optogenetic activation of OptoH_3_R in vivo produces long-term effects on animal behavior or neuronal population activity remains unexplored. Given that H_3_R primarily functions as a presynaptic autoreceptor regulating histamine and other neurotransmitter release, future validation in living animals combined with behavioral paradigms and fiber photometry would be of greater translational value. Collectively, our findings establish OptoH_3_R as a mimic of endogenous H_3_R β-arrestin signaling. This optogenetic tool provides a valuable platform for interrogating the physiological and pathophysiological roles of H_3_R with high spatiotemporal resolution, particularly in complex neuronal circuits where histamine release patterns are highly dynamic.

## Data Availability

All data generated and analyzed during the current study are available from the corresponding author on reasonable request.
